# Neuroprotection Offered by Majun Khadar, a Traditional Unani Medicine, during Cerebral Ischemic Damage in Rats

**DOI:** 10.1093/ecam/nep224

**Published:** 2011-06-05

**Authors:** Seema Yousuf, Fahim Atif, Muzamil Ahmad, Tauheed Ishrat, Badruzzaman Khan, Fakhrul Islam

**Affiliations:** ^1^Neurotoxicology Laboratory, Department of Medical Elementology and Toxicology, Jamia Hamdard (Hamdard University), New Delhi 110062, India; ^2^Brain Research Laboratory, Department of Emergency Medicine, Emory University, Atlanta, Georgia 30322, USA

## Abstract

Stroke results in damages to many biochemical, molecular and behavioral deficits. Present study provides evidence of the protective efficacy of a Unani herbal medicine, Majun Khadar (MK), against cerebral ischemia-induced behavioral dysfunctions and neurochemical alterations in the hippocampus (HIP). Transient focal cerebral ischemia was induced for 2 h followed by reperfusion for 22 h in a rat model. Rats were divided into four groups: sham, middle cerebral artery occluded (MCAO), drug sham (MK; 0.816 g kg^−1^ orally for 15 days) and MK pre-treated ischemic group (MK + MCAO). Levels of enzymatic and non-enzymatic antioxidants were estimated in HIP along with behavioral testing. MK pre-treatment significantly (*P* < .05–.001) restored the activities of glutathione peroxidase (GP×), glutathione reductase (GR), glutathione S-transferase (GST) and decreased the level of lipid peroxidation (LPO) and H_2_O_2_ content in HIP in the MK + MCAO group which were severely altered in the MCAO group. The content of glutathione (GSH), total thiols (TT) and ascorbic acid (AsA) was significantly depleted in the MCAO group; pretreatment with MK was able to restore its levels. Also in the MK + MCAO group, significant (*P* < .5–.001) recovery in behavioral testing by rota rod and open-field activities was seen as compared with the MCAO group. MK alone did not show any change neither in the status of various antioxidants nor behavioral functions over sham values. Although detailed studies are required for the evaluation of exact neuroprotective mechanism of MK against cerebral ischemia these preliminary experimental findings conclude that MK exhibits neuroprotective effect in cerebral ischemia by potentiating the antioxidant defense system of the brain.

## 1. Introduction

A 100% prophylaxis over cerebral ischemia-induced neuronal death which initiates intracellular acidosis, increased concentrations of intracellular calcium and overproduction of reactive oxygen species is still a long way to come. Ischemic damage is severe in those regions where cerebral blood flow is restored because reflow to previous ischemic brain regions results in an increase in the oxygen level; therefore, severe oxidative injury occurs [1]. The penumbral region of the ischemic territory has the potential to be salvaged by timely drug intervention.

Growing attention is being paid to traditional medicines, as they have been proved therapeutically/prophylactically fruitful against manifold diseases, which can be evaluated by the dependence of 3.2 billion people (64%) of the whole world population on traditional medicines [2]. Many workers reported the beneficial effects of traditional medicines against brain ischemia. Yousuf et al. [3, 4], while evaluating the effect of Khamira Abresham, Majun Baladar against cerebral ischemia-induced oxidative damage in discrete brain part, report a protective role of these Unani formulations by the synergistic modulation of its various antioxidant compounds. Numerous single compounds have also shown their importance, for example, Saffron and herbal extract of *Nardostachys jatamansi* were also found to be protective against cerebral ischemic oxidative damage [5, 6]. Ahmad et al. [7] reported the neuroprotection offered by sesame oil on brain hippocampus after focal cerebral ischemic damage.

**Figure 1:**
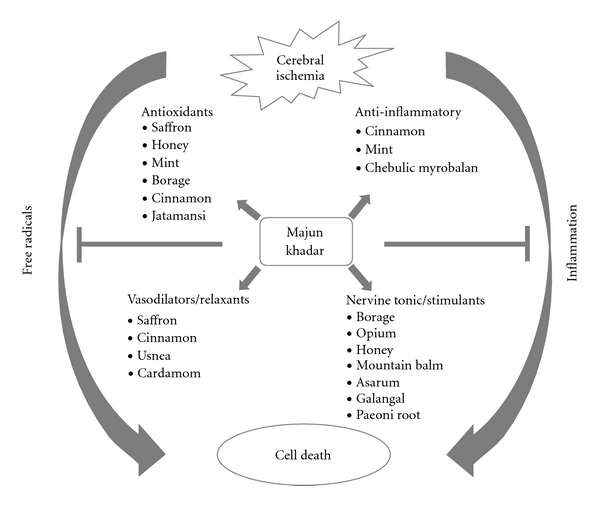
Hypothetical presentation of possible mechanisms of action of Majun Khadar (MK) against cerebral ischemia. Free radicals and inflammation are two major precipitators of ischemic cell death. Different constituents of MK have antioxidant and anti-inflammatory properties and many of them act as vasodilator, relaxant, nervine tonic and nerve stimulant which directly or indirectly prevent ischemic damage.

The promising results of Khamira Abresham and Majun Baladar prompted us to evaluate the neuroprotective efficacy of another Unani herbal formulation, Majun Khadar (MK), against cerebral ischemic damage. MK has 27 constituents, with different therapeutic values and has firm grounds of pharmacological efficacy in disorders ranging from heart, nerve, gastrointestinal disorders, and so forth [8, 9]. These constituents of MK individually are potent free radical scavengers (Figure 1) (saffron, borage, mint, cinnamon, *Nardostachys jatamansi*); anti-inflammatory and brain boosting agents (cinnamon, honey, chebulic, myrobalan, mint); and vasodilators/nerve relaxants (cardamom, usnea, saffron) (see Table 1 for references). Since the oxidative stress and inflammation play a key role in ischemic neuronal death, we evaluated the synergistic efficacy of these different constituents by testing MK against cerebral ischemia-induced oxidative damage in rat brain and associated behavioral dysfunctions. 

## 2. Methods

### 2.1. Drugs and Chemicals

Glutathione (oxidized and reduced), nicotinamide adenine dinucleotide phosphate-reduced form (NADPH), Oubain, Phenol Red, Horseradish peroxide (Hrp), 1-chloro-2,4-dinitrobenzene (CDNB), 5,5′-dithiobis-2-nitrobenzoic acid (DTNB) and thiobarbituric acid (TBA) were purchased from Sigma-Aldrich (St. Louis, MO, USA). Other chemicals were of analytical reagent grade. Majun Khadar (MK) was procured from Hamdard (Waqf) Laboratories (Ghaziabad, Uttar Pradesh, India).

### 2.2. Animals

Male Wistar rats, weighing 300–350 g, were used for the study. Rats were obtained from the Central Animal House of Jamia Hamdard, New Delhi, India. They were housed in polypropylene cages in air-conditioned room and allowed free access to pellet diet and water *ad libitum*. The studies were carried out in accordance with the official regulations approved by the Animal Ethics Committee of Jamia Hamdard, New Delhi, India.

### 2.3. Experimental Protocol

The animals were divided into four groups (*n* = 6). The first group served as sham and saline was given orally. The second group was the ischemic group (MCAO: middle cerebral artery occluded) in which ischemia was induced for 2 hours followed by reperfusion for 22 hours. The third group was pre-treated with MK (0.816 g kg^−1^, orally for 15 days, single dose daily) followed by ischemia/reperfusion (MCAO + MK group). The fourth group was pre-treated with MK alone for 15 days as a drug control. After the last dose of MK was given on day 15, ischemia/reperfusion was induced on the same day. After reperfusion, the animals were assessed for neurobehavioral activities and then sacrificed.

### 2.4. Induction of Ischemia

The right middle cerebral artery occlusion was produced using an intraluminal filament model as earlier described by Longa et al. [11]. In brief, the rats were anesthetized with chloral hydrate (400 mg kg^−1^, intraperitoneally). A 4-0 nylon monofilament pre-coated with poly-l-lysine was inserted into the external carotid artery and advanced into the internal carotid artery. Two hours after the induction of ischemia, the filament was slowly withdrawn and animals were then returned to their cages for a period of 22 hours of reperfusion. In the sham-operated rats, the external carotid artery was surgically prepared for insertion of the filament but the filament was not inserted. The body temperature of animals was maintained with an infrared lamp and was continuously monitored for respiration and unresponsiveness throughout the surgical procedure. No animal mortality was observed during experimental procedures.

### 2.5. Behavioral Study

The behavioral tasks adopted in this study were designed to assess impairments consistent with the known functional architecture of the rat brain. The behavioral tests in each group were performed and the studies were carried out between 9:00 a.m. to 4:00 p.m. under standard laboratory conditions. All tests were performed and analyzed by subject blind to the experiment.

### 2.6. Physical Performance Tests

#### 2.6.1. Rota-Rod (Motor Coordination)

Omni rotor (Omnitech Electronics Inc., Columbus, OH, USA) was used to evaluate the motor in-coordination. It consisted of a rotating rod (75 mm diameter), which was divided into four compartments, permitting test of four rats at a time. The apparatus automatically recorded time 0.1 s, when the rats fall off the rotating shaft. The speed was set at 10 rpm and the cut-off time was 180 s.

#### 2.6.2. Open-Field Tests (Locomotor Activity)

Open-field tests were done according to the method of Yamamoto et al. [12] with slight modification at ambient temperature ranging between 26 and 32°C. The mean of session totals of vehicle and treatment groups were compared for locomotion (s), rest (s), distance travelled (cm), average speed (cm s^−1^). Videopath analyser (Coulbourn Instrument, Allentown, PA, USA) consists of an open-field chamber (50 × 50 × 35 cm), and a video camera was fixed over the chamber by an adjacent rod, an activity monitor, a programmer and a printer. The session total for all parameters was taken. Observations were recorded for 10 min.

### 2.7. Tissue Preparation

After producing MCAO and assessment of behavioral parameters, the animals were sacrificed immediately and their brains were taken out to dissect the hippocampus (HIP). Post-mitochondrial supernatant (PMS) obtained from 10% homogenate of tissue was used for the estimation of various parameters related with oxidative stress.

### 2.8. Biochemical Estimations

#### 2.8.1. Lipid Peroxidation (LPO)

The procedure of Utley et al. [13] was used for the estimation of lipid peroxidation with some modifications. One milliliter of homogenate (2.5% in chilled KCl) was pipetted in a 20 ml glass tube and incubated at 37 ± 1°C in a metabolic shaker for 60 minutes. Similarly, 1 ml of the same homogenate was pipetted in a centrifuge tube and incubated at 0°C. After 1 hour of incubation, 1.0 ml 5% chilled tetrachloroacetic acid was added followed by 1.0 ml of 0.67% TBA in each vial and proper mixing was done after each addition. The aliquot from each vial was transferred to a centrifuge tube and centrifuged at 2000× g for 15 minutes. Thereafter, the supernatant was transferred to another tube and placed in the boiling water bath. After 10 minutes, the test tubes were cooled and the absorbance of each aliquot was measured at 535 nm. The rate of lipid peroxidation was expressed as nanomoles of TBA reactive substance formed/hours/g of tissue using a molar extinction coefficient of 1.56 × 10^5^ per M per cm.

#### 2.8.2. Glutathione (GSH)

Reduced glutathione was determined by the method of Jollow et al. [14]. Post-mitochondrial supernatant was precipitated with sulfosalicylic acid (4.0%) in the ratio of 1 : 1. The samples were kept at 4°C for 1 hour and then centrifuged at 1200 × g for 15 minutes at 4°C. The assay mixture contained supernatant, 0.1 M phosphate buffer and DTNB (stock = 100 mM in 0.1 M phosphate buffer) in a total volume of 3 ml. The optical density of the reaction product was read at *λ* 412 nm and results were expressed as nmol GSH/g tissue.

#### 2.8.3. Estimation of Total Thiols (TT)

The assay was done according to the method of Sedlak and Lindsay [15]. The assay mixture contained 0.2 ml of homogenate (10% w/v), 0.1 M Tris-EDTA (pH 8.2) and 1.0 mM DTNB in total volume of 2.7 ml and mixed well. Thereafter, 8.3 ml methanol was added and centrifuged at 3500 × g for 5 minutes. The yellow color developed was read immediately at 412 nm in a spectrophotometer (UV-1601, Shimadzu, Japan). The total thiol content was calculated by using a molar extinction co-efficient of 13.6 × 10^3^ M^−1^ cm^−1^.

#### 2.8.4. Glutathione Peroxidase (GPx)

The activity of GPx was assayed according to the method described by Mohandas et al. [16]. The reaction mixture (2 ml) consisted of a 1.44 ml sodium-phosphate buffer (pH 7.4), 0.1 ml EDTA (1 mM), 0.1 ml of 1 mM sodium azide, 50 *μ*l glutathione reductase (1 IU ml^−1^), 0.1 ml of 1 mM GSH, 0.1 ml NADPH (0.2 mM), 0.1 ml of 10% PMS and 10 *μ*l of 0.25 mM H_2_O_2_. The enzyme activity was calculated as nmol NADPH oxidized/minutes/mg protein, using a molar extinction co-efficient of 6.22 × 10^3^ M^−1^ cm^−1^.

#### 2.8.5. Glutathione S-Transferase (GST)

The assay for the activity of GST was measured as described by Habig et al. [17]. For the assay of GST with CDNB as the substrate, the reaction mixture (2 ml) consisted of a phosphate buffer (0.1 M, pH 7.4), 1 mM GSH, 10 mM CDNB and 10% post-mitochondrial supernatant. An increase in optical density at 340 nm was recorded every 30 s for 3 minutes. The results were calculated by using the molar extinction co-efficient of 9.6 × 10^3^ M^−1^ cm^−1^ for CDNB conjugate at 340 nm. The enzyme activity was expressed as nmol of CDNB conjugate formed/minutes/mg protein.

#### 2.8.6. Glutathione Reductase (GR)

The activity was measured by the method of Carlberg and Mannervick [18]. Briefly, the reaction mixture (2 ml) consisted of 1.60 ml of phosphate buffer (0.1 mM, pH 7.4), 0.1 ml NADPH (0.1 mM), 0.1 ml EDTA (0.5 mM), 0.1 ml GSSG (1 mM) and 0.1 ml of post-mitochondrial supernatant (10%). The reaction mixture was incubated at 30°C for 5 minutes before adding post-mitochondrial supernatant. The enzyme activity was determined by measuring the disappearance of NADPH at 340 nm using an extinction co-efficient 6.22 × 10^3^ M^−1^ cm^−1^. Specific activity of the enzyme was expressed as NADPH oxidized/minutes/mg protein.

#### 2.8.7. Catalase (CAT)

Catalase was estimated by the method of Claiborne et al. [19]. The assay mixture consisted of 1.95 ml of 0.1 M phosphate buffer (pH 7.4), 50 *μ*l of post-mitochondrial supernatant (10%). The reaction was initiated by adding 1.0 ml of 0.019 M hydrogen peroxide. A decrease in optical density at 240 nm was recorded after every 30 s for 3 minutes. The enzyme activity was calculated in terms of nmol H_2_O_2_ consumed/minutes/mg protein.

#### 2.8.8. Na^+^K^+^ATPase

The activity of Na^+^K^+^-ATPase was determined as inorganic phosphorous (pi) release, using the method of Svoboda and Mossiger [20]. The Na^+^K^+^-ATPase activity was determined in two reaction media, A and B. The reaction mixture A consisted of 0.2 M KCl, 1.0 M NaCl, 0.1 M MgCl_2_, 0.2 M Tris-HCl buffer, pH 7.4, and 0.1 ml of brain homogenate (10%) in a total volume of 2.0 ml. The reaction mixture B consisted of 0.1 M MgCl_2_, 10 mM ouabain, 1.0 M NaCl, 0.2 M Tris-HCl buffer, pH 7.4, and 0.1 ml of brain homogenate (10%) in a total volume of 2.0 ml. The enzyme reaction was started by adding 0.2 ml of 25.0 mM ATP at 37°C and terminated after 15 minutes by adding 1.0 ml chilled 10% TCA. The mixture was centrifuged and the supernatant (0.5 ml) was used for the estimation of inorganic phosphorous according to method of Fiske and Subborow [21].

#### 2.8.9. Assay for Hydrogen Peroxide (H_2_O_2_)

Hydrogen peroxide was assayed by H_2_O_2_-mediated horse-radish peroxide (Hrp)-dependent oxidation of phenol red by the method of Pick and Keisari [22] with slight modification. 0.2 ml microsomes were suspended in 1.0 ml of solution containing 0.28 nM phenol red, 8.5 units Hrp, 5.5 mM dextrose and 0.05 M phosphate buffer (pH 7.0) incubated at 37°C for 60 minutes. The reaction was stopped by the addition of 10 ml of NaOH (10 N), and then centrifuged at 800 g for 5 minutes. The absorbance of the supernatant was measured at 610 nm against reagent blank. The quantity of H_2_O_2_ produced, expressed as nmol H_2_O_2_/h/g tissue based on the standard curve of 50–250 nM H_2_O_2_-oxidized phenol red.

#### 2.8.10. Ascorbic Acid (AsA)

The ascorbic acid content was estimated by the method of Majhi et al. [23]. The reaction mixture consisted of 0.5 ml of 2–4 dinitrophenyl hydrazine reagent (prepared in 1 M HCl) and 0.1 ml of supernatant obtained after precipitation with 20% TCA. The mixture was incubated at 60°C for 1 hour. After cooling 2.5 ml of 8.5% H_2_SO_4_ was added while the tubes were in ice. The absorbance was taken at 540 nm and the results were expressed as mg ASA/g tissue.

### 2.9. Statistical Analysis of Data

Data were analyzed for significance of difference using the Student's *t*-test. Values are expressed as means ± SE (*n* = 6). The significance of results was ascertained at *P* < .05.

## 3. Results

### 3.1. GSH, AsA and TT

**Figure 2:**
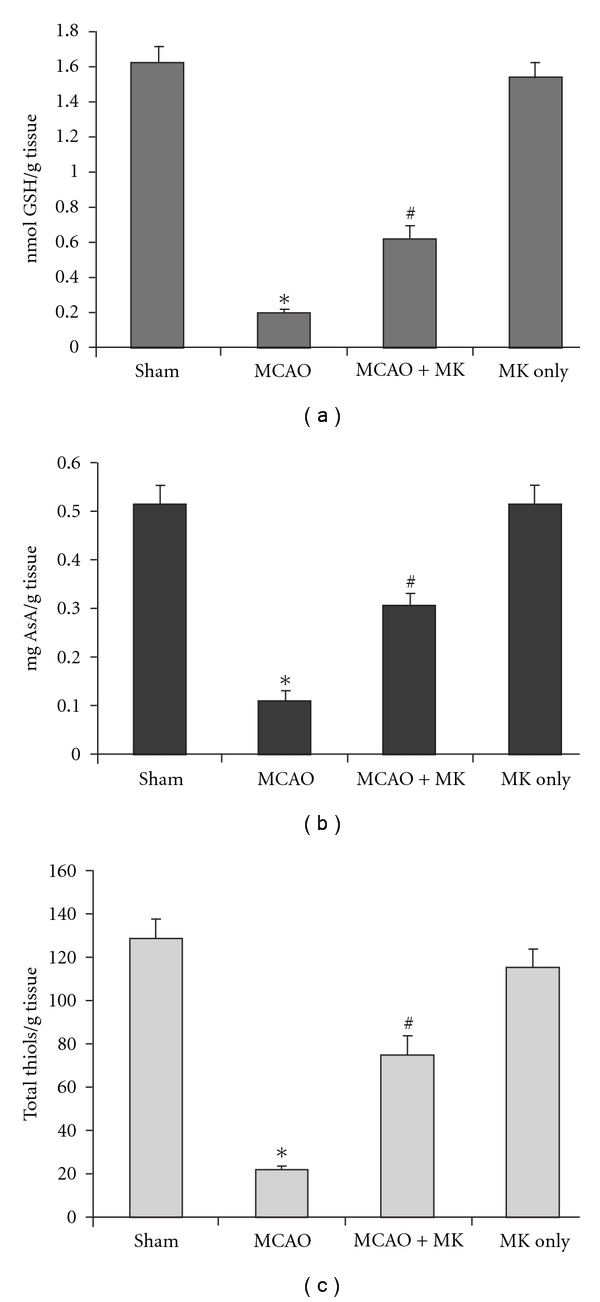
GSH content (a), ascorbic acid (b) and total thiols (c) in the hippocampus. Values are expressed as means ± SE (*n* = 6). Significance was determined as *P < .001 when compared with sham; ^#^ *P* < .01 as compared with the MCAO group.

A significant decrease (*P* < .001) in the status of various endogenous cellular antioxidants, GSH, AsA and TT homeostasis was observed in the MCAO group when compared with the sham group (Figures 2(a), 2(b), and 2(c)). MK pre-treatment exhibited a significant restoration (*P* < .01) in GSH, AsA and TT content in the MCAO + MK group when compared with the MCAO group (Figures 2(a), 2(b) and 2(c)). The MK sham group did not alter the status of GSH, AsA and TT as compared with sham values. 

### 3.2. Antioxidant Enzymes and Na^+^K^+^ATPase

Activities of various antioxidant enzymes and Na^+^K^+^ATPase in the hippocampal region of different groups have been listed in Table 2. The activity of GPx, GR, GST, CAT and Na^+^K^+^ATPase was significantly depleted (*P* < .001) in the MCAO group when compared with the sham group values. Whereas in the MCAO + MK group, MK pre-treatment showed a significant (*P* < .05–.01) restoration in the level of various enzyme and Na^+^K^+^ATPase as compared with MCAO group. MK alone did not show any change in the status of any enzyme over control values.

### 3.3. LPO and H_2_O_2_ Content

**Figure 3:**
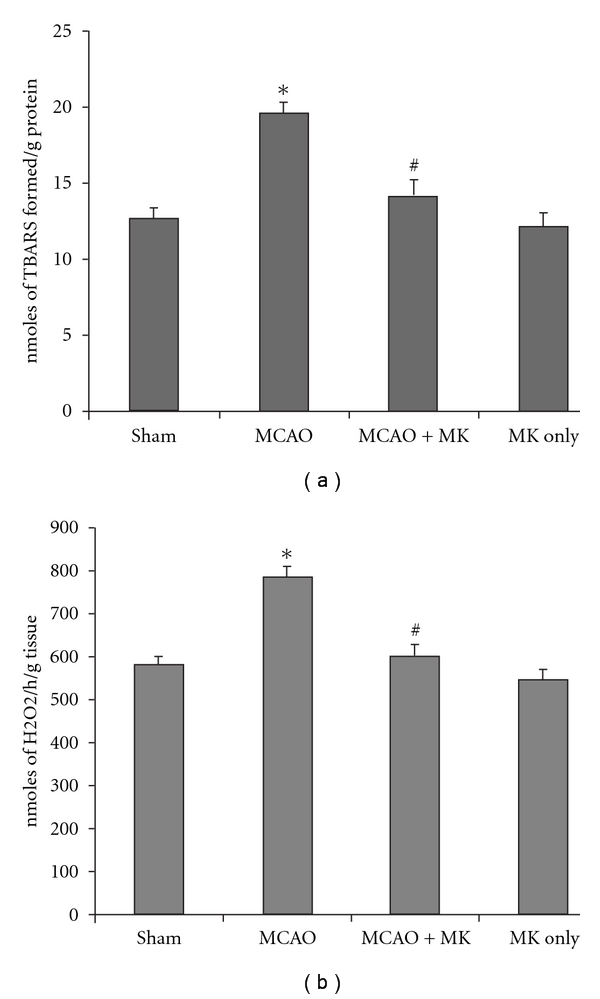
LPO level (a) and H_2_O_2_ content in different groups in hippocampus. Values are expressed as means SE (*n* = 6). Significant difference *P < .001 when compared with sham values; ^#^ *P* < .01 when compared with the MCAO group.

The level of LPO and H_2_O_2_ content adds to the proof of the loss of scavengers and increased peroxidative damage during cerebral ischemia. A significant increase (*P* < .001) in the content of LPO and H_2_O_2_ was observed in the MCAO group when compared with the sham group. In the MCAO + MK group, a significant decrease (*P* < .01) was seen in the level of LPO and H_2_O_2_ when compared with the MCAO group (Figures 3(a) and 3(b)). The MK sham group did not show any significant alterations in the status of TBARS and H_2_O_2_ when compared with the sham group. 

### 3.4. Motor Coordination

**Figure 4:**
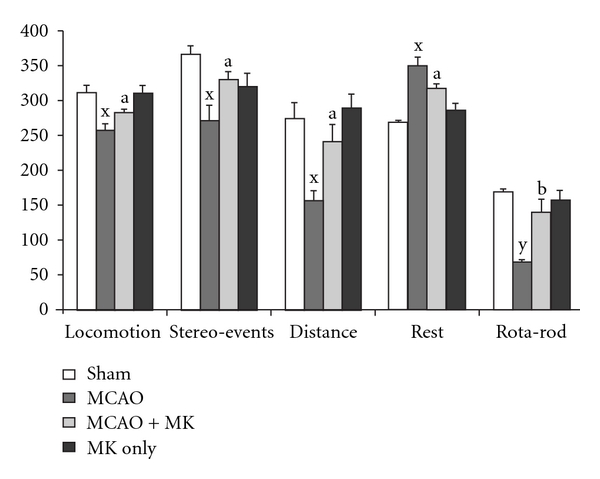
Neurobehavioral activities (locomotion, stereoevents, distance traveled, rest and rota-rod) in various groups. Significance was determined as ^x^ *P* < .01; ^y^ *P* < .001 when compared with sham values and ^a^ *P* < .05; ^b^ *P* < .01 when compared with the MCAO group.

Significant impairments were observed at the behavioral outcomes in the ischemic group. On the rota-rod task, a test that requires good coordination, balance and sensorimotor function, the MCAO group showed significant (*P* < .001) impairments in coordination and balance as compared with the sham animals. Although they appeared to be motivated to run on the rod, rats with stroke could not keep up with the acceleration of the rod and fell approximately twice than control rat. But significant (*P* < .05) improvement was observed in the MCAO + MK group in staying on the accelerating rod for a longer duration of time when compared with the MCAO group (Figure 4). 

Open-field activity was related to ipsilateral cerebral hemisphere volume and the motor measurements. Open-field activities (locomotion, stereo events, distances traveled, average speed and rest) showed significant (*P* < .01) deficits in the MCAO group when compared with the sham group. Whereas in the MCAO + MK group, the activities were reinstated significantly (*P* < .05) when compared with the MCAO group (Figure 4).

## 4. Discussion

In this study, supplementation of MK for 15 days significantly boosted the neurobehavioral outcomes and neuronal defense mechanism against cerebral ischemia by increasing antioxidants activity related to lesion pathogenesis. The recuperation of the brain from oxidative stress during reperfusion after ischemia was preceded by recovery of GSH with its dependent enzymes and decreased oxidative damage. Restoration of the antioxidant homeostasis in the brain after reperfusion may have helped the brain recover from ischemic injury. Although MK is extensively used in Unani medicine, it lacks scientific grounds for its efficacy and to the best of our knowledge this is the first study to report its possible protective mechanisms against cerebral ischemic damage. One of the universally accepted etiologies of stroke is imbalance between factors of free radical formation and the maintenance of the neuronal integrity through the endogenous defense mechanism. In the present study, the major cellular defenses (both enzymatic and non enzymatic) were severely altered in the ischemic group as compared with the sham group, which was due to the increased formation of reactive oxygen species (ROS) and dwindling levels of the radical scavengers. However, this imbalance was prevented by MK supplementation. A substantial elevation in the LPO level and H_2_O_2_ content along with the depletion in the activity of various protective antioxidant enzymes in the ischemic brains which has been validated with immense earlier reports was observed [3, 4, 24, 25]. However, pre-treatment with MK resulted in the reversal of the increased LPO level, H_2_O_2_ content and up-regulated the depleted antioxidant enzymes when compared with the ischemic group suggesting the decreased formation of ROS or radical scavenging activity of MK after ischemic injury. Neuronal and cognitive impairments were seen to be improved after stroke with decoction of herbs (Gagamjungjihwan) and (Frustus Euodiae) and the Chinese herbal formula (FBD) by the restoration of GSH content and lowering the lipid peroxides during ischemic damage [26, 27].

Diminished supply of oxygen and energy to the brain leads toward the first step of oxidative damage. A drug having vasodilatory action may be the answer for this problem that might increase the oxygen and energy supply. The neuroprotection offered by MK may be due to the additive effect of the principle components, for example, saffron, honey, *Nardostachys jatamansi*, cardamom, mint, cinnamon, black pepper, mastich and usnea. Saffron and its active component, crocetin has antioxidant potential [28, 29], lipid lowering and vasodilatory properties which leads to increased blood flow thereby supplying oxygen and nutrient to the ischemic penumbra [30]. Honey is a well-known antioxidant, anti-microbial, wound healing and nutritive agent with its final stamp for accreditation from US Medical Archives. The presence of various polyphenolic compounds, pinocembrin, pinobanksin, chrysin, galagin, ascorbic acid, enzymes (glucose oxidase, catalase and peroxidases) attribute to its antioxidant potential [31, 32]. In the present study, increased activity of antioxidant enzymes and less peroxidative damage to the brain can be attributed to the presence of honey in MK. MK also contains *Nardostachys jatamansi* which has the potential to scavenge superoxide radicals, hydroxyl radicals and singlet oxygen [33, 34] due to which it was able to offer protection during cerebral ischemic damage. Malva et al. [35] indicated its neuroprotective property against signaling pathways involving (Ca^2+^)_i_ and the redox state of the cells, which can play its role in ischemic damage. The mechanism by which *Nardostachys jatamansi* exerts its effect is not clearly known but it enhances the function of gamma amino butyric acid (GABA) [36–38] and GABA potentiates the treatment of ischemic stroke [39] because there are increased concentrations of excitatory amino acids. These properties of *Nardostachys jatamansi* potentiates the antioxidant and neuroprotective potential of MK against cerebral ischemic damage. The presence of mint another important constituent in MK adds to the anti-inflammatory, antioxidant and/or radical scavenging properties [40–43]. The presence of carveol, an active organic constituent of mint, and transition elements (e.g., Mn, Zn and Co) is responsible for the free radical scavenging activity of the mint [44]. All these properties might have synergistically led to decreased ischemic damage after MK pre-treatment.

Variant behavioral tasks were employed in the present study to detect motor and sensorimotor dysfunction in rats after cerebral ischemia. We observed significant alterations in the motor, locomotion, rest time and stereo events in the ischemic group when compared with the sham group. But in the MK pre-treated group, a positive restoration in the mentioned behavioral events was observed when compared with its respective controls. This amelioration in behavior may be attributed to the protection offered by its various pharmacologically important constituents of MK such as *Nardostachys jatamansi*, cinnamon, saffron and mint. It might be due to antioxidant potential of *Nardostachys jatamansi* that may have helped to restore the antioxidant levels which play an important role in improving the behavior. Various reports suggest the improvement in behavioral outputs offered by *Nardostachys jatamansi* [45, 46]; our data are in agreement with these previous reports. The vasodilatory activity of carotenoids present in saffron, antioxidant potential of mint and brain-boosting function of cinnamon that enhance cognition, working memory, visual-motor speed added to the effectiveness of MK against cerebral ischemia-induced behavioral dysfunctions [47]. The collective properties of various constituents of MK such as increasing blood flow, oxygen content, radical scavenging and brain boosting actions may have restored the damaged regions of the brain and thereby improving the behavioral outcomes. Again a Chinese herbal medicine, Tokishakuyakusan, reported to suppress impairments of lower limbs and influencing visuospatial perception for post-stroke patients [48].

Exploring the intricate protection offered by MK may bring healthy, safe, natural, multimodal protective properties of these various plant extracts which can help to protect the brain from ischemic damage. In conclusion, MK has anti-ischemic properties and it protects brain from cerebral ischemia-induced oxidative damage due to the additive effect of its various pharmacologically important constituents that possess antioxidant, ROS scavenging and vasodilatory properties. Apart from the above-mentioned efficacies, it would be very interesting to explore specifically any possible neuro-nutrient role of MK in neurological disorders as it has seven constituents that are nerve boosters.

## Funding

Central Council for Research in Unani Medicine (CCRUM), Govt of India, New Delhi.

## Figures and Tables

**Table 1:** Constituents of *Majun Khadar* (MK).

S.N.	Urdu/Arabic name	English name	Origin	Content (w/kg)	Clinical Importance
(l)	Mastagi	Mastich	Plant/gum	2 g	Stimulant, catarrh, astringent [10].
(2)	Boazyadan	Sweet pelitory (*Pyrethrum indicum* Linn)	Plant/leaves/seed/oil	2 g	Weakness of nerves, Arthritis, Gout, overall body strengthener
(3)	Shakakal	Shekakul (*Pustinaca Secacut L*)	Plant/Root	2 g	Sexual debilities
(4)	Bheman	Behen (*Centaurea behen* Linn)	Plant/Root	2 g	Cardiotonic, relaxant, palpitation, delirium, sexual debilities [10].
(5)	Gaozaban	Borage (*Borage Officinalis Linn*)	Plant/leaves/flowers	2 g	Cardiotonic, nervine tonic, palpitation, weakness of brain, pneumonia, T.B, Infectious diseases [10].
(6)	Badranjboya	Mountain Balm	Plant/leaves	2 g	Strengthens heart, exhilarant, melancholia, nervine tonic [10].
(7)	Sumbul-Teeb	Valerian (*Valeriana Officinalis Linn*)	Plant/Root	2 g	Antispasmodic, epilepsy, hysteria, bronchitis, loss of appetite, liver disorders [10].
(8)	Aushna	Usnea (*Usnea longissima Linn*) (Old man's beard)	Plant/leaves	2 g	Cardiotonic, palpitation relaxant, delirium [10].
(9)	Qust-Shireen	Kartas root	Plant/Root	2 g	Respiratory diseases, G.I.T disorders [10].
(10)	Elaichi	Cardamom	Plant/Fruit	2 g	Vasodilator, Digestive, Diuretic, Cardiotonic, Stimulant, carminative, anti-flatulent, cure for obesity [10].
(11)	Barg-Faranjamshak	Ram Tulsi (Sweet Basil)	Plant/Seed/leaves	2 g	Cardiotonic G.I.T disorders [8].
(12)	Saad-kofi	Sipertenius	Plant	2 g	Cardiotonic, nervine tonic, weakness of heart, weakness of brain, G.I.T disorders, liver diseases [8].
(13)	Uood-Saleeb	Paeoni Root (*Paeonia officinalis*)	Plant/Root	3 g	Epilepsy, paralysis, facial paralysis, meningities [10]
(14)	Dalchini	Cinnamon (*Cinnamomum verum)*	Plant/Bark	3 g	Gastrointestinal, antibacterial, carminataive, stimulant, Antiflatulant, cardiotonic, gastritis [10].
(I5)	Halhiya Kabuli	Chebulic Myrobalan (Terminalis chebula)	Plant/Bark/leaves/ Fruit	4 g	Nervine tonic, memory enhancer, stomach & intestine protectant, eye-sight enhancer, laxative, blood purifier, cures bleeding, ulceration of gums [10].
(16)	Takhm Khaskhas Safeed	Poppy plant (*Papaver somniferum* Linn)	Plant/Seed	4 g	Analgesic, Protects digestive system [10]
(17)	Falfol daraz	Long pepper (*Piper longum* Linn)	Plant/Fruit	7 g	Nerve depressant, Cough, asthama, whooping cough [10].
(18)	Falfal Siyah	Black pepper (*Piper nigrum* Linn)	Plant/Fruit	7 g	Digestive, expectorant, general body strengthener [10].
(19)	Darong Akrabi	Leopard's bane (*Doronicum hookarii* Linn)	Plant/Root	7 g	General body strengthener, cardiotonic, delirium, paralysis [10].
(20)	Sajaz Hindi	Cinnamon Tamala	Plant/Roots/Wood	7 g	Cardiotonic, weakness of heart, hypertension, weakness of brain, antispasmodic [10].
(21)	Shahed	Honey	Animal		Antibacterial, antibiotic
(22)	Pudina	Mint	Plant/Leaves	7 g	Digestive, anti-emetic, Beauty packs [10]
(23)	Asaroon	Asarum (Asarum ewrospaeum Linn)	Plant/Root/Leaves	7 g	Nervine tonic, paralysis [10].
(24)	Shoranjan	Colchicum (Colchicuni autumale L.)	Plant/Bulbs	4 g	Arthritis [10].
(25)	Khoalnajan	Galangal (*Alpinia galangal wild*)	Plant/Roots	2 g	General body strengthner, Expectorant, Nervine tonic [10].
(26)	Zafran	Saffron	Plant/Flower	1.5 g	Strengthner, antioxidant [10].

**Table 2:** Effect of cerebral ischemia on the activity of various enzymes in the hippocampus.

Enzymes	SHAM	MCAO	MCAO + MK	MK ONLY
GP×	16.30 ± 0.93	7.59 ± 0.52^a^	12.86 ± 1.22^x^	16.00 ± 1.02
		(53.43%)	(69.43%)	(−1.84%)
GR	34.54 ± 1.50	21.65 ± 1.04^a^	27.81 ± 1.06^x^	33.70 ± 1.41
		(37.31%)	(28.45%)	(−2.43%)
GST	16.29 ± 0.72	9.37 ± 0.63^a^	12.58 ± 0.98^y^	16.92 ± 0.8l
		(42.48%)	(34.25%)	(15.83%)
CAT	6.38 ± 0.33	3.23 ± 0.21^a^	4.86 ± 0.38^y^	5.37 ± 0.41
		(49.37%)	(50.46%)	(−15.83%)
Na^+^K^+^	4.52 ± 0.25	2.21 ± 0.13^a^	3.46 ± 0.26^x^	4.21 ± 0.24
ATPase		(51.10%)	(56.56%)	(−6.8%)

Values are expressed as mean ± S.E (*n* = 6) in nmoles/minutes/mg protein. Values in parentheses show the percentage increase or decrease with respect to their control. Significance was determined as ^a^ *P* < .001 sham versus MCAO. ^x^ *P* < .01; ^y^ *P* < .05 MCAO versus MCAO + MK.
